# Soft Pneumatic Actuator Fascicles for High Force and Reliability

**DOI:** 10.1089/soro.2016.0029

**Published:** 2017-03-01

**Authors:** Matthew A. Robertson, Hamed Sadeghi, Juan Manuel Florez, Jamie Paik

**Affiliations:** Reconfigurable Robotics Laboratory (RRL), École Polytechnique Fédérale de Lausanne (EPFL), Lausanne, Switzerland.

**Keywords:** artificial muscles, soft actuators, biomimetics, modular systems, wearable robots

## Abstract

Soft pneumatic actuators (SPAs) are found in mobile robots, assistive wearable devices, and rehabilitative technologies. While soft actuators have been one of the most crucial elements of technology leading the development of the soft robotics field, they fall short of force output and bandwidth requirements for many tasks. In addition, other general problems remain open, including robustness, controllability, and repeatability. The *SPA-pack* architecture presented here aims to satisfy these standards of reliability crucial to the field of soft robotics, while also improving the basic performance capabilities of SPAs by borrowing advantages leveraged ubiquitously in biology; namely, the structured parallel arrangement of lower power actuators to form the basis of a larger and more powerful actuator module. An SPA-pack module consisting of a number of smaller SPAs will be studied using an analytical model and physical prototype. Experimental measurements show an SPA pack to generate over 112 N linear force, while the model indicates the benefit of parallel actuator grouping over a geometrically equivalent single SPA scale as an increasing function of the number of individual actuators in the group. For a module of four actuators, a 23% increase in force production over a volumetrically equivalent single SPA is predicted and validated, while further gains appear possible up to 50%. These findings affirm the advantage of utilizing a fascicle structure for high-performance soft robotic applications over existing monolithic SPA designs. An example of high-performance soft robotic platform will be presented to demonstrate the capability of SPA-pack modules in a complete and functional system.

## Introduction

Soft pneumatic actuators (SPAs), which are intrinsically compliant and readily manufacturable, are paving the way for a future of new robotic systems that benefit from inherent safety, adaptability, and customizability.^1–4^ While the force output of a robotic actuator is an important performance metric for many applications, it does not always accurately reflect the requirements of a full robotic system in others. Among other common performance standards, robustness and controllability stand out as key features that should not be underestimated in the development of a reliable robotic device. A common method for improving the robustness of some engineered systems is the introduction of a fail-safe or a secondary mechanism that can be activated in the case of unexpected primary mechanism failure.^5^ In some systems, this fail-safe operation may be an emergency shut-off or a similar irreversible action, while in others it may be accomplished by the addition of redundancy or parallel multiplicity. In the biological world, this is demonstrated in many different domains. Certain insects are capable of maintaining mobility despite the loss of nearly any number of their legs,^6^ and larger order animals’ muscular tissue, essential for maintaining nearly all activities of daily living, is arranged with parallel cellular structures that serve the dual purpose of redundancy and the summation of parallel action to increase strength.^7^ This latter example is as applicable to engineered systems, such as robots, as it is to biology.

The concept of grouping smaller actuators to form larger ones is not uncommon in nature, as muscular tissue makes use of this to a great effect. The strategy is likewise not uncommon in the world of robotics and even within the realm of soft actuation. Attempts have been made to utilize conventional pneumatic artificial muscles (PAMs), such as the well-known McKibben type of SPA in a bunched parallel configuration, but they have not been without difficulty imposed by interference between the individual actuators.^8^ PAMs feature an inflatable elastomer core surrounded by a braided fiber outer sheath,^8,9^ which expands radially when activated. This expansion is orthogonally coupled to the actuation stroke and consequently does not allow the necessary freedom of movement between individual fascicles of these actuators in a close group when inflated. Parallel grouping of McKibben actuators was explored in Pritts and Rahn^10^ for the primary purpose of achieving multi-degree-of-freedom (DoF) activation of continuum robot segments rather than force enhancement alone, but avoided the issue of internal actuator interference by providing adequate spacing between artificial muscle units. In the biological analog, muscles evade the issue, in part, because they are inherently a form of “wet-ware,” being self-contained in a self-lubricating, fluidic environment that allows muscle fibers to move freely among each other while still combining their output effort. A related concept demonstrated the use of a specific “functional arrangement” of multiple PAM units embedded in a compliant matrix to enforce actuator separation and constraint.^11^ In this latter case, multidimensional actuator grouping was used to achieve a soft actuated material with programmable multi-DoF motion.

Soft actuator literature is predominantly concentrated on single actuators instead of arrays of multiple units. Soft actuation technology such as shape memory material (shape memory polymers [SMP], shape memory alloys [SMA]), electro active polymers, and fluidic elastomer actuators has drawn attention for soft systems,^1,12–14^ but SPAs are still the most common actuation choice for applications where intrinsic safety and adaptability are needed. A variety of soft actuated wearable hand exoskeleton and glove devices have been developed to leverage the adaptability, natural bending motion, and relative strength of SPAs for assistive and rehabilitation applications.^15–19^ A large body of work also targets augmentation of lower extremity muscle and joint work to improve healthy normal functionality for walking or restore and assist deficiencies in gait dynamics.^20–24^ Compared to previous exoskeletons engineered for these tasks, these soft-actuated devices are remarkably lower in weight, inertia, and mechanical impedance, and consequently very well suited to wearability in both structured therapeutic environments and in daily living.

Although the precise construction of SPAs can vary, they are commonly found in the form of a slender, thin-walled, hollow, and cylindrical structure externally constrained by inextensible fibers and driven by a readily available pressurized air supply. The actuators are activated by applying positive relative pressure to the hollow internal cavity and can be designed to produce linear,^15^ bending,^25,26^ and torsion forces,^27^ or even any combination thereof,^28^ by including restraining features,^29^ or by selecting an appropriate angle of constraining fibers relative to the direction of applied motion or force. As a result of their fundamentally simple construction, SPAs have already been used in a broad range of applications, including rescue robots,^30^ rehabilitation,^20,21^ bioinspired robotics,^31^ and wearable technologies.^16,18,19,32,33^ This is due, in part, because they are highly customizable, easy to fabricate, lightweight, and low cost.^34^

While the straightforward design of individual SPAs is not amenable to generating the typically high forces required of traditional robotic applications or human body-scale wearable devices, SPAs are a notably attractive candidate for use in the development of an alternative type of high-force soft actuator composed of a parallel grouped assembly of smaller SPAs. The simple and self-contained design of single SPA units facilitates a novel method of integration to enable functional grouping of SPAs. Short of mimicking biological architecture directly, the SPA pack developed here draws inspiration from the idea of “constrained isolation” necessary for utilizing actuators in parallel—keeping each actuator effectively free to move independently, while sufficiently and not overly bounded.

## Objective

In this work, a unit fiber-reinforced, linear-extending SPA will first be produced as the basis for a larger parallel-pack configuration. A four-unit pack (*4Pack*) will then be fabricated and characterized to demonstrate and motivate the principles of the design architecture. To show the effectiveness of the new SPA concept in application, a high-force multi-DoF platform actuated by SPA packs will be introduced and briefly studied. Finally, a discussion will include a summary of the primary benefits of the SPA-pack configuration and comparison to current alternatives, extensibility of the actuator modules to further applications, and some logistics of a more independent architecture for control and power distribution.

• Novel SPA-pack structure suited for fiber-reinforced SPAs, which demonstrates improved robustness and reliability over individual SPAs.
• Fiber-reinforced SPA design for high-force applications above 100 N.
• Prototype and experimental validation of an active, multi-DoF platform powered by high-force SPA packs under closed loop control.

## Materials and Methods

### New SPA concept: SPA packs

By grouping SPAs in parallel, actuator packs can be formed, which outperform individual SPAs of comparable size. Not only can higher force output be achieved with this configuration but also the benefit mutually increases with the number of constitutive actuator units utilized in the pack. This is to say that the more unit actuators are combined in parallel, the greater the gain in force output can be attained. This improvement in spatial-force efficiency allows for either stronger actuation in a given physical space or more compact actuation for a given required force. These features hold considerable value as they extend the design space within the soft robotics domain.

Beyond the improvement of existing performance metrics over volumetrically equivalent single actuators, the SPA packs also introduce new measures of performance not realizable without the grouped configuration. Robustness and reliability are improved as a result of the inherently modular pack structure. In the event of an SPA unit failure within the pack, the parallel actuator is still capable of functioning at an only slightly reduced capacity. In certain use cases, there may even be no decrease in functionality at all, as a unit actuator loss may be compensated by shifting work (increasing pressure) to the remaining units in the pack. This extends the utility of soft actuators in robotics even further to applications, where safety not only benefits from the use of compliant materials but also from the improved controllability of devices powered by soft actuator packs.

**FIG. 1.**
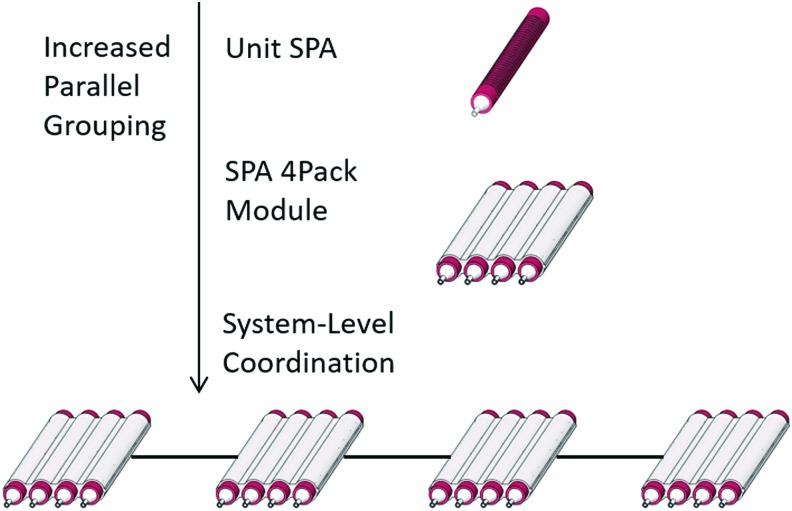
Extension of SPA modularity. Parallel grouping of SPA packs enables system-level high performance that can be utilized for wearable applications or high-force multi-DoF soft actuation. DoF, degree-of-freedom; SPA, soft pneumatic actuator. Color images available online at www.liebertpub.com/soro

The simple nature of this concept also reflects no dependency on pack structure beyond the parallel arrangement of the grouped actuators, enabling multiple possible form factors to be considered for a given required force output. This design freedom allows for highly customizable actuator units. The variability in structure can be used to accommodate applications with different size or geometric requirements. Furthermore, the concept of design modularity can be extended not only to the actuators themselves but also equally to the applications, where high-performance packs are particularly well suited, such as the realm of wearable robotics. Taking advantage of this modularity, parallel groups of SPA packs may be configured to produce further cumulative output, as well as the potential for utilizing individual packs to generate directional, multi-DoF actuation at the system level. Figure 1 illustrates the evolution of the SPA as a modular base unit, giving rise to high-performance soft robotic systems.

### SPA-pack fabrication

A single-unit SPA is first fabricated for use as the basis of the SPA pack. The individual actuator is designed to produce linear extension force and displacement, with a high-angle fiber wrapped around a single-chamber hollow elastic body. The inextensible fiber acts to restrict the radial inflation and expansion of the actuator, while allowing it to extend in length as pressure is applied to the internal cavity. PAM actuators make use of a relatively low fiber angle (<54.7°),^9^ to produce high-tensile force. However, it has also been demonstrated that at higher angle fiber arrangements, extension and torsion forces can be achieved.^35^ To allow operation at high pressure, a smooth geometry is used along the surface of the inner chamber with no sharp internal corners to minimize the stress concentration as the actuator swells. The fiber angle set at roughly 75° relative to the longitudinal axis of the actuator is chosen deliberately to be above the zero force threshold (54.7°) derived from a simplified analytical actuator force model, shown in Equation (1), introduced in Chou and Hannaford^9^ to produce a linear extension motion and low enough to allow easily repeatable and consistent wraps of the fiber, which is wound onto the actuator manually.

The hollow elastic body of the actuator constructed here is manufactured following a series of conventional and lost wax casting methods. A solid wax core is constrained by a 4-mm-diameter metal rod at the center of a rigid, hollow, ABS plastic mold fabricated rapidly by 3D FDM printing, into which uncured silicone rubber (Elastosil^®^M4601) is poured between the core and mold. The chosen material is appropriately designated to allow flexibility (up to 700% elongation at break) as well as strength (6.5 N/mm^2^) for use with high pressure.^36^ On the outer surface of the overall cylindrical structure of the actuator body, a row of raised bumps is also formed from the body mold, which is used to accurately align and guide consistent wraps of a nylon fiber wound four times along the length in alternating directions. After the silicone rubber is allowed to cure at room temperature for 12 h, the actuator is removed from the mold and wrapped with the outer fiber before removing the rigid wax core. Once the outer fiber has been wrapped and secured with a silicone-based adhesive (SilPoxy^®^), the internal wax core is heated in an oven above its melting point and poured out with the supporting rod removed. Additional SilPoxy is used to attach silicone pneumatic supply tubes at one end of the actuator and to seal the opposite end. The resulting structure is a completed single-unit linear SPA. The dimensions of the final SPA unit are 145 mm in length and 17 mm in diameter.

Each unit SPA in a pack is held together by a soft connecting interstitial membrane and behaves the same as an individual SPA, despite being bonded together. The highly elastic membrane easily stretches in response to the output force of each actuator, and relative motion between actuators from uneven pressure distribution or inconsistencies from fabrication is accommodated. While providing the necessary and subtle bond to hold the SPA pack together as one module, the interstitial membrane also provides another important function, enforcing adequate spacing between unit SPAs to eliminate sliding friction and interference effects. This flexible constraint enables each actuator unit in the pack to function individually uninhibited, while still acting together as a functional group.

**FIG. 2.**
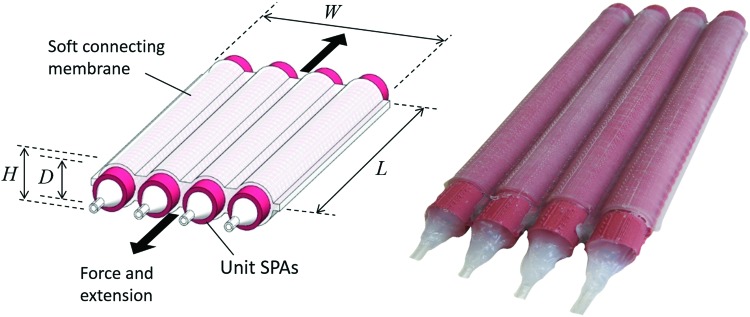
Diagram and photograph of 4Pack actuator. The dimensional parameters given in Table 1 are illustrated with the basic components and operating mode of a four unit SPA pack. Color images available online at www.liebertpub.com/soro

The fabrication of an SPA pack, shown in Figure 2, follows a basic casting procedure to integrate four partially completed unit SPAs. Before the wax cores are melted out, individual actuators are inlayed between a 3D-printed ABS and a two-piece mold, which allows for empty space, surrounding the central segment of the actuators. Uncured low-elastic modulus silicone rubber (Ecoflex^®^00-30) is then poured into the mold to fill the space and subsequently bond the unit SPAs together within a shared flexible membrane housing. After curing at room temperature for 4 h, the SPA pack is removed from the mold. The pack is placed in an oven to allow the wax cores to melt for removal. SilPoxy is then used to attach a silicone tube to each SPA unit in the pack and seal the opposite end, as in the case of the individual actuators.

The simplicity of this actuator design enables the application of a likewise simple analytical model to predict output force performance. While this model is developed in literature for another type of tensile soft actuator, it is equally applicable to the extension type studied here.

### Actuator analytical model

A simple analytical model can be used to estimate the force output of a single-unit SPA, shown in Equation (1).^9^ For this kinematic model, the actuator is represented as a perfect hollow cylinder with wall thickness, *t_k_*, and assumptions that the system is lossless and without energy storage. Furthermore, this model is not effective for describing quasistatic or dynamic characteristics of fiber-reinforced pneumatic actuators. Although noncomprehensive, this model nonetheless provides a sufficient result for use in comparison between different SPAs. While the result will not be used to describe the best theoretical fit to the experimental performance of an SPA with given parameters, it is still expected to provide a reasonably close approximation of blocked force capacity, where actuator elongation is restricted. The simplified static model of force output *F* for a fiber-reinforced SPA is given as follows:

\begin{align*}
F = {{{P^\prime} {b^2} \left( {3{ \rm{co}}{{ \rm{s}}^2} \theta -
1} \right) } \over {4 \pi {N^2}}} + \pi P^\prime \left[ {{b \over
{N \pi }}{t_k} \left( {2 \sin \theta - {1 \over { \sin \theta }}}
\right) - t_k^2} \right] \tag{1}
\end{align*}

where,

\begin{align*}
b = \sqrt {{L^2} + {{ \left( {D \pi N} \right) }^2}} \tag{2}
\end{align*}

and *L* is the actuator length, *D* is the cylindrical diameter, $\theta$ is the angle of a single wrapped reinforcing fiber relative to the axial direction, *N* is the number of wraps of the fiber around the actuator body, and *b* is the length of the fiber shown in Equation (2) as a function of actuator dimensions and the number of fiber wraps. The pressure ${P^\prime} = P - {P_{atm}}$ is the pressure applied to the internal chamber relative to atmospheric pressure ${P_{atm}}$, with *P* as the absolute internal pressure. For the parameters given in Table 1, and depicted in Figure 2, the corresponding theoretical output force of a single-unit SPA is expected to be 30.5 N. In addition to predicting the output for unit SPAs, this basic model can also be used to extrapolate and anticipate the effect of parallel actuator grouping, as well as for comparison of such actuator packs to equivalent monolithic SPA designs.

**Table 1.** Parameters Given for Fabricated Actuators and Their Estimated Force Output

*Parameter*	*Value*
${P^\prime}$	200 kPa
${P_{atm}}$	101,325 kPa
*L*	145 mm
*D*	17 mm
$\theta$	75.2°
*N*	16 wraps

## Results

### SPA packs versus single SPAs

Estimating the output of an SPA pack in a parallel fascicle arrangement following the simple unit SPA model is trivial under the assumption that the soft interstitial membrane connecting the units does not store energy or add significant impedance to the pack system. The output is therefore the total theoretical output of individual SPA units, modeled by Equation (1), multiplied by the number of units in the pack. In the example SPA pack fabricated here, the number of units is 4, so the total force output is expected to be $4F$. Again using the parameters, given in Table 1, this simple model yields an estimate of 122 N per SPA pack.

Using the force model for a single SPA, it is also possible to estimate the performance of actuators volumetrically equivalent to an SPA *n*Pack, where *n* is the number of unit actuators contained in the pack. For a cross-sectional area, *A,* of an individual unit actuator, the “equivalent SPA” is defined to have equal volume to a pack of *n* unit actuators, by defining its cross-sectional area as *A_eq_ = nA*. For a pack of *n* = 4 actuators, the area of an equivalent SPA is then held to be *A_eq_ = 4A*. This simple relationship for the equivalent area defines the necessary diameter for an equivalent single SPA. All other actuator parameters, as shown in Table 1, are held equal.

**FIG. 3.**
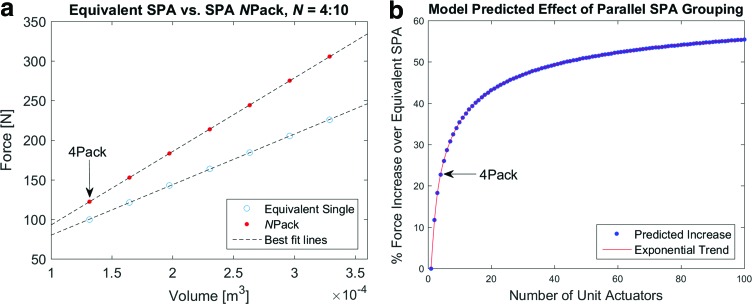
Comparison of SPA pack to Equivalent SPA. **(a)** For comparable volume and the same construction parameters, an SPA pack produces a larger force output than an equivalent single SPA. As the number of individual units in an SPA pack increases, the difference calculated as a percentage also increases following the trend shown in **(b)**. Color images available online at www.liebertpub.com/soro

Within the range plotted in Figure 3(a), the model follows a primarily linear trend with a consistently greater force output by the SPA pack, in comparison to an equivalent SPA. The divergent slopes between the SPA pack and an equivalent SPA force output suggest an increase in the gain of grouped actuator output over the corresponding equivalent SPA as the number of actuator units in the pack increases. Looking further, it is apparent from Figure 3(b) that the trend is in fact bounded, but up to and above the range of unit actuators shown indicates a diminishing return that may be inefficient as well as impractical in real use. The optimal number of units for a specified volume to maximize spatial-force efficiency of an SPA pack in this model framework will not be investigated in this study, but it may be observed that using 20 or fewer unit SPAs in an actuator pack, which is subjectively manageable under the paradigm of current popular SPA manufacturing methods, can still yield a theoretical gain up to 40%.

From this analytical model investigation, it is found that the gain in the predicted performance of actuators in a grouped parallel configuration offers an additional advantage to the improved robustness and modularity afforded by the architecture, which can be seen as a significant benefit of SPA packs over single SPAs of equivalent volume. The result of the spatial efficiency dictated by this force–volume relationship can be leveraged to design higher force actuators within a given space, or smaller actuators for a prescribed force. Regardless of the utilization, however, the unique, parallel fascicle arrangement of SPAs presented here enables a new alternative to high-force soft actuation.

### SPA-pack characterization and application

The SPA pack described here previously is evaluated experimentally for characterization as well as comparison, with the simplified force output models presented above. Given a maximum force output of 122 N estimated by the model, the linear SPA pack is tested using a single-axis load cell, which can measure forces up to 200 N (BCM 166H). In addition, the test setup consists of two rigid outer supports, a pressure regulator, a data acquisition system, and a pneumatic solenoid control valve. The experimental test results yield a maximum output force of 112 N per module, roughly 8.2% lower than the expected value.

**FIG. 4.**
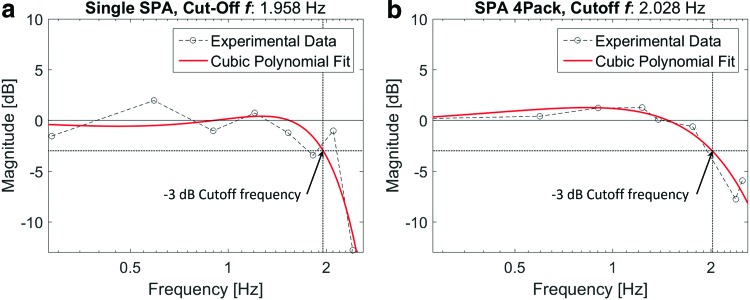
Frequency response of individual SPA and 4Pack. The corresponding force output of an individual SPA, and a pack of four unit actuators to a sinusoidal pressure input is tested at multiple frequencies to obtain the bode diagrams above. These diagrams indicate a cutoff frequency of nearly 2 Hz for both the individual actuator **(a)** and the 4Pack **(b)**, and no loss of bandwidth performance from parallel actuator grouping. The figures do qualitatively indicate an increase in the damping ratio of an actuator pack compared to a single SPA, by the apparent “flattening” of the frequency response; however, this was not calculated. Color images available online at www.liebertpub.com/soro

To determine the bandwidth of the SPAs, a sinusoidal input pressure with amplitude of 1 bar is applied to the unit and module at varying frequencies. In Figure 4, the frequency response to the sinusoidal input reveals the −3 dB bandwidth of both the individual SPA and the pack module to be around 2 Hz. The consistency in the measured bandwidth between the single- and parallel-grouped actuators indicates that the flexible constraint used to assemble the SPA pack does not interfere with the performance contributed by each of the individual internal actuators.

**FIG. 5.**
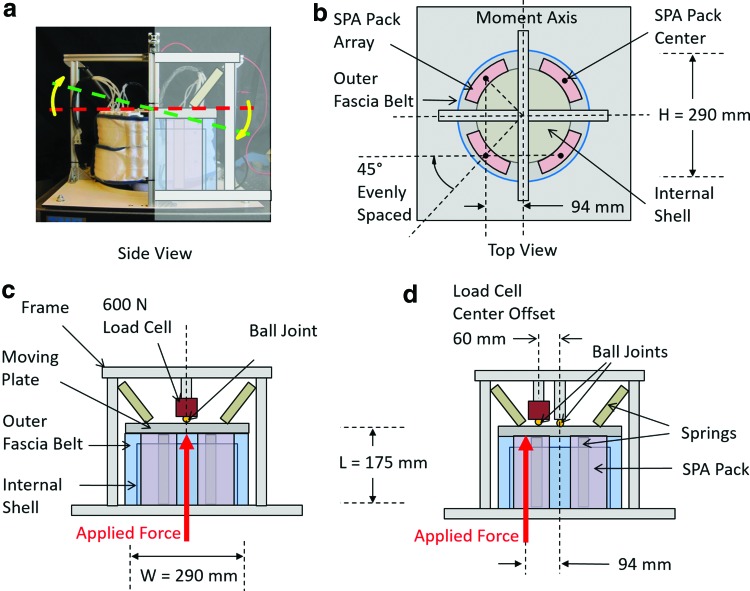
High-force soft actuated platform. Photograph **(a)** depicts the actual multi-DoF platform surrounded by a rigid aluminum frame, along with a depiction of the angular DoF used in moment measurement. Diagram **(b)** illustrates the configuration of the platform from a *top* view, showing the concentric array of SPA packs. The blocked force measurement configuration **(c)** is used to measure total vertical force capacity of the actuated platform. The blocked moment measurement configuration shown in **(d)** is used to measure the maximum moment applicable on the pitch axis by two synchronized modules, as well as to measure the moment frequency response of the system. The *curved arrows* indicate the direction of angular displacement upon activation from rest at horizontal. Color images available online at www.liebertpub.com/soro

To demonstrate the integration of the high-force actuator packs developed here in a complete robotic system, an active, multi-DoF platform was constructed and tested. The platform and test fixture were fabricated using rigid aluminum plates and an extruded aluminum frame. The fixed base section of the platform comprised a 3D-printed ABS plastic shell, around which a modular, reconfigurable circular array of SPA packs are constrained laterally by 3D-printed locating features, and held in place radially by a flexible fabric belt to restrict the motion of the actuators to their respective linear output axis. The actuator configuration is depicted in the top view shown in Figure 5(b). The upper moving section of the test setup consists of a plate suspended from above and below by springs mounted to the frame, which enables preloaded degrees of freedom in all directions.

The performance of the SPA prototype was evaluated using the test setup shown in Figure 5(a) designed to simulate basic concentric geometric and multiaxial loading conditions. The SPA packs in the prototype are connected to a benchtop electro-pneumatic system and controlled directly from a virtual instrument in LabVIEW, through National Instruments cRIO-9082 integrated real-time controller with a 1.33-GHz dual-core processor and an LX150 FPGA. An ITV1031-21F1N-Q pressure regulator (SMC) enables digital control of the pressure supplied to each SPA up to 5 bars, and one solenoid valve per pack module is used to enable or disable the SPA packs independently.

The test setup was reconfigured for three different system measurements. To measure the maximum vertical force that can be applied by the platform, a 600 N-range, single-axis load cell mounted to the frame was positioned at the center of the moving upper plate. A ball joint was utilized at the interface of the load cell with the plate to eliminate moment transmission and effectively measure a pure point force. The configuration is illustrated in Figure 5(c). All four SPA packs in the surrounding of the platform were activated simultaneously at maximum operating pressure (2 bars) to steady state at the full extension. The resulting blocked force on the load cell was recorded to be greater than 432 N. In addition, the preloaded upper plate of the actuator platform was measured alone to apply 36 N of force at the position of actuator module maximum extension, such that the cumulative force capacity of the actuator modules independent of the platform is the sum of these forces, for a total force of 468 N. This value is 4.1% lower than the expected force capacity of 488 N predicted by the model and based on the simple additive effect of actuator packs working in parallel.

A similar configuration was used to measure the maximum moment applied by the SPA platform, as shown in Figure 5(d). The load cell used to measure blocked force was relocated to a position between the axis of rotation at the center of the plate and the location of the vertical actuator force, to a radial distance of 60 mm from the center of the plate. Two actuator packs applied force at 94 mm from the center of the plate to produce the maximum moment from the given configuration and the number of packs in the system. Again, the load cell was mounted to the frame rigidly, and force from the plate was applied through a ball joint to record only force at a point. To simplify measurement and isolate the rotational behavior from linear actuation, the center of the moving upper plate was constrained relative to the frame using a ball joint. The principle axis of the moment applied by the SPA packs was then assumed to be orthogonal to the radius of action, where the linear force was applied. The maximum moment applied was thus calculated from the blocked force measurement from the load cell and the known location of the applied constraint force, yielding a blocked moment of nearly 18 N-m along the radial pitch axis of the platform.

**FIG. 6.**
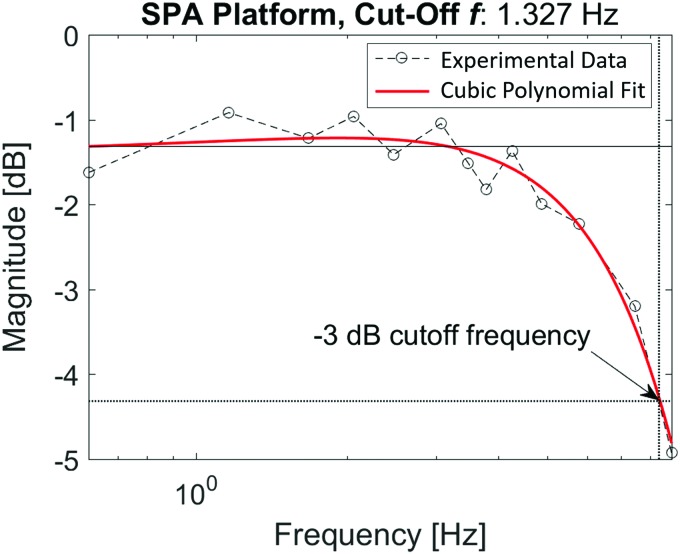
Frequency response of the high-force SPA platform. The plot shows the magnitude of moment response to a sinusoidal pressure input for a spectrum of frequencies from which the system cutoff frequency at −3 dB is found to be 1.33 Hz. Color images available online at www.liebertpub.com/soro

The measure of system bandwidth was conducted using the blocked moment measurement configuration. A sinusoidal pressure input was used to actuate the platform, while the resulting moment output was recorded by the load cell. The amplitude of the resulting sinusoidal moment output of the plate was compared to the amplitude of the input sinusoid for various frequencies to create a frequency response diagram shown in Figure 6, from which the bandwidth of the SPA-pack array and test platform system was calculated to be 1.33 Hz.

The characteristics of the SPA-based, high-force, multi-DoF actuated platform developed and measured here are summarized in Table 2 below. In addition, characteristics of the constitutive actuators are also included in the table for comparison and summary.

**Table 2.** Summary of Modular SPA Characteristics

	*Single*	*4Pack*	*Multi-DoF platform*
Dimensions (W × H × L) or (D × L), mm	17 × 175	84 × 19 × 175	290 × 290 × 175
Weight, g	28	185	945
Maximum load force capacity, N	26	112	468
Bandwidth, Hz	1.958	2.028	1.327
Maximum moment capacity, N-m	—	—	18.00
Angular range of motion, °	—	—	±5

DoF, degree-of-freedom.

While the highest force measured for an SPA module under individual testing only reached 112 N, the inconsistency between that and the average force per module calculated from the platform testing of 117 N can be attributed to the difference in actuator constraints used during each measurement. This also likely contributed to the slight difference between bandwidth measurements for the single and 4Pack modules, which were calculated from their respective force outputs. In single-module testing, deflection caused by buckling of the flat actuator plane was restricted using rigid, immovable parallel plates on both sides of the module to enable a purely linear reaction against a single-axis load cell. A similar effort was made in the multi-DoF platform to apply the necessary constraint to the modules while also allowing linear extension of the actuator with minimal impedance, but in this case, a flexible outer fascia belt was used instead of a fixed rigid constraint on the outer side of the modules. The outer belt provided radial constraint but was not fixed to the grounded structure, and free to move linearly in the directions of force application with the actuators. This additional freedom reduced the impedance to the actuators yielding an average force per module when tested as part of the multi-DoF platform of roughly 4% above the individually measured 4Pack, and nearly 4% below the output predicted by the analytical model used in the Materials and Methods section. Similarly, higher force measurements were attained for the SPA-pack module in comparison to a single actuator during blocked force testing as a result of buckling in two dimensions, which was even more difficult to constrain.

### Reliability testing

**FIG. 7.**
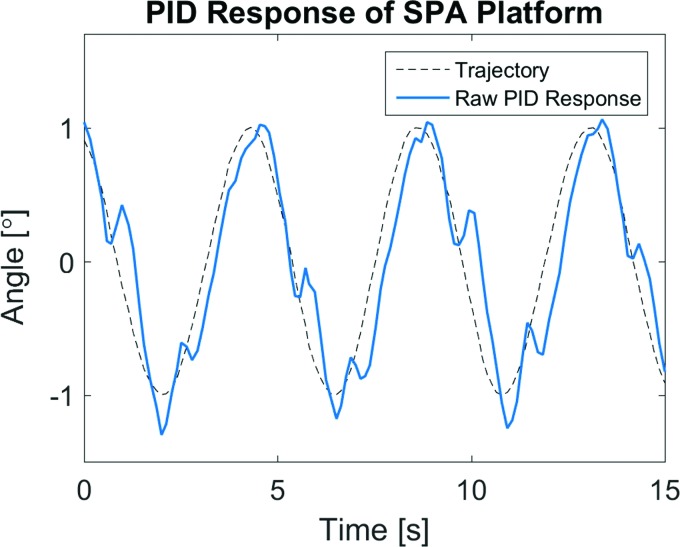
Closed loop control of quadruple SPA-pack platform. The actuated platform follows a sinusoidal trajectory under proportional-integral-derivative (PID) control, with transient disturbances evident from discretized control logic imposed by antagonistic pairing of unidirectional actuator modules. Color images available online at www.liebertpub.com/soro

The multi-DoF robotic system was also tested with a proportional-integral-derivative (PID) controller following a sinusoidal trajectory of the platform angle, measured using an inertial measurement unit (IMU) mounted on the upper plate. The controller follows the desired trajectory, as seen in Figure 7, but suffers from hardware limitations imposed by the unidirectional actuators, and a consequently discretized control strategy. With antagonistic pairing of actuator pack modules, only one of each opposing actuator in the circular SPA-pack array is operated at a time, dictated by the direction of motion required. Switching between these modes, namely between positive and negative angular motion, adds an inherent delay determined by the control system hardware that includes an IMU, solenoid valves, data acquisition (DAQ), and a laptop computer communicating over multiple USB peripherals, in addition to the dynamics of the actuator units themselves. This results in sharp overshoot upon mode switching, visible in Figure 7 as short twitches in the measured angle. Regardless, closed loop control of the actuated platform is achieved and the raw response shown in the figure below.

**FIG. 8.**
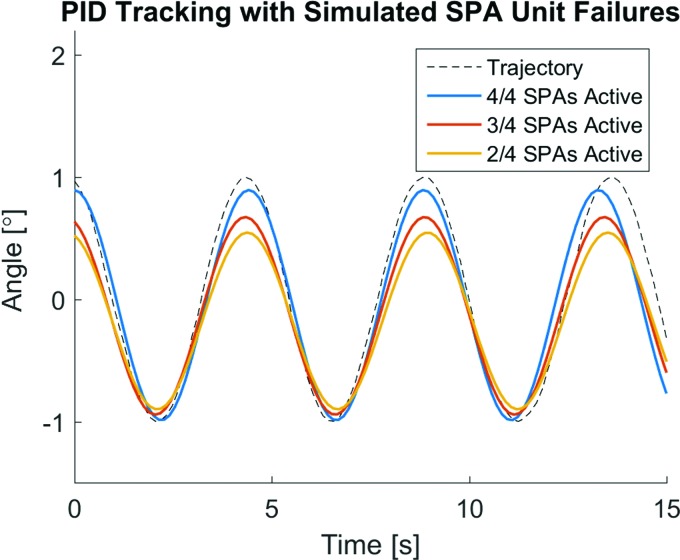
Demonstration of quadruple SPA-pack robustness. The closed loop response of the SPA-pack actuated robotic platform following a sinusoidal trajectory shows reliable stability and controllability with different numbers of active units, despite an asymmetric drop in performance. Color images available online at www.liebertpub.com/soro

As a demonstration of fascicle structure robustness to individual actuator unit malfunction or degradation, the SPA platform is tested with simulated failures by cutting off the air supply to different numbers of units in a given module. The mock failure is induced reversibly by tightly pinching the silicone pneumatic supply tube to individual units in the pack, using a plastic locking zip tie. Reversal of the action is accomplished by cutting the plastic tie without damaging the flexible supply tube to restore the particular unit to full functionality to allow iterative testing. Figure 8 shows that as individual actuators in a pack are cut off from the controlled air supply, the platform is able to maintain reduced capacity tracking on the affected side and nearly unchanged tracking on the unaffected side. Overall, the platform also maintains general controllability despite the loss of any number of units in the pack (with the clear exception of losing every unit in the pack, not shown). Sinusoidal fits were made to the controlled response data to more clearly represent the subsequent change in the platform behavior to actuator reduction.

Given the repeated structure of independent constitutive elements composing SPA packs, the actuator modules of parallel grouped SPAs exhibit a capacity for reliability and robustness against failure modes common to any engineered system. As safety is regarded a leading promise of soft robotics, these qualities elect the SPA-pack architecture studied here to be among the most essential elements of technology for use in future applications.

## Discussion

SPA packs can be used to create soft robotic systems with high-performance capabilities. In much the same way that conventional SPAs are integrated into active, multi-DoF applications, these actuator modules utilize standard electro-pneumatic control systems to deliver high force and reliability for a newly accessible range of tasks. The soft actuated platform constructed in the Results section generated 18 N-m of moment and demonstrated cumulative vertical force production of 468 N, corresponding to an average output of 117 N per actuator pack. An open loop system bandwidth over 1.3 Hz was recorded for the multi-DoF platform, while the bandwidth of a separately tested SPA 4Pack and individual unit SPA was found to be 2 Hz. While the frequency response independently measured for the actuators showed no significant effect from the soft interstitial coupling used to fabricate the pack, the additional dynamics from integration in the multi-DoF platform contributed to the reduction in overall system bandwidth. Due to the repeated, parallel structure of smaller SPA units, the SPA packs also demonstrated a unique robustness to potential failures, by permitting the loss of one or several units without an overall loss in functionality of the actuator group or system at large under closed loop control.

It is shown in the Results section that a *fascicle arrangement of SPAs is capable of generating more linear force than an equivalent single SPA of comparable volume.* Moreover, the gain in force production continues to increase within practical bounds as the number of units in a parallel configured SPA pack is increased. This model-based finding indicates that high-performance SPA design favors multiplicity and can be exploited as a new actuator design strategy, which takes into consideration the effect of multiple unit actuators in parallel coordination as well as the individual unit actuator design parameters to achieve desired performance. Although extensive effort was not made to ensure the absolute accuracy of the kinematic analytical model used for this analysis as introduced in the Materials and Methods section, validation was achieved by comparison of actual actuator pack force measurement to the model-predicted value. The measured result of an SPA 4Pack performance was found to be 4% lower than expected. Better results in future work might be obtained either by use of a dynamic model,^37^ or through finite element analysis tools for SPAs.^29^

As it is shown and discussed in Ref.,^35^ varying the fiber angle of fiber-reinforced SPAs can yield either extension or contractile behavior. As seen in Pritts and Rahn,^10^ the nonlinear relationship between fiber angle and force output is different above and below this functional fiber angle threshold of 54.7°, dictated by the model shown in Chou and Hannaford.^9^ Before this study, it has not been shown that higher fiber angle, extension-type SPAs can be used to perform in the range of force output over 100 N. Between the two generalized regimes of SPA fiber arrangement, from 0° to 54.7°, and from 54.7° to 90°, only those in the lower angle category have demonstrated performance up to this force magnitude. As this work has successfully shown, high fiber angle SPAs offer an intrinsically safe, soft actuation alternative for high-performance applications when used in modular, parallel groups. Furthermore, the architecture affords reliability and customizability not readily available from other soft actuation technologies.

Further work remains to implement true autonomous reliability of SPA packs. As designed and tested here, each of the unit actuators within the fabricated SPA packs is operated from a shared air supply, such that the fail-safe and robust benefit of multiplicity can only be demonstrated by manual isolation of a simulated “failed” unit from the supply. While the units in a pack are designed to activate together, in which case a shared supply is appropriate, the core intention of parallel actuators is to enable independent failure, where a shared supply then becomes a shared burden. The simplest solution is to utilize individual solenoid control valves per each unit actuator in a pack. While direct, however, this may not represent the most optimal use of component size or weight, as in normal operation most valves in a pack will be activated simultaneously, and redundantly. Alternatively, the addition of passive, pneumatic logic gates may be incorporated directly to future designs of the individual SPA units to reduce the number of independent control solenoid valves needed. Such passive valves may allow air to flow in and out of an individual actuator in the presence of an appropriate pressure differential, but cut off the supply mechanically and automatically in the event of a unit actuator failure.

As SPAs advance toward use in increasingly complex multi-DoF soft robotic systems, the complexity of the soft pneumatic subsystems required to operate them is also expected to follow. While the problems inherent in the logistics and control design of conventional complex pneumatic systems have been addressed to the point that there exist commercially available solutions, these have not been well defined in the world of soft robotics, where weight, size, and power are an objective concern. Off-the-shelf pressure regulators, valve manifolds, integrated sensors, and electronic controllers are built for industrial scales and consequently not readily appropriate for many applications where SPAs best serve. In this regard, it is therefore worth noting that at this point, the future development of soft robotics may not be entirely restricted to the development of better actuators alone, but in the integration of actuator module purpose built for robotic applications. Although the SPA-pack design presented here incorporates only morphological changes to accomplish readily beneficial progress toward robot-centered soft pneumatic systems, future work may also include the evolution of SPA modules with embedded sensing, control, or even low-level processing capabilities to further enforce the concept of their modular utility.

## Conclusion

We proposed a design method to address one of the major challenges of current soft robotics: shortcomings of soft actuators. By strategic actuator grouping, we have demonstrated with a prototype that we can augment mechanical performance and reliability in soft actuators. Diverting from single SPAs and adopting the use of SPA-pack modules afford several useful benefits. The first, maybe most obligatory, benefit is that of increased force production. Biological muscles are named and denoted singularly following a simple notion that an arrangement of multiple single actuators in a group outperforms multiple scaled single actuators: they in fact comprised infinitesimal smaller actuators at the cellular level. In this view, it is then no less acceptable to produce a higher performance SPA module by parallel configuration of smaller SPAs. Efforts have been initiated to demonstrate this effect with PAM actuators but with limited success owed to interference between closely grouped actuators caused by the coupled relationship of actuator bulging on inflation to force production. Here we demonstrated that the concept extends more elegantly to SPA-type actuators. These actuators by comparison do not expand significantly on inflation and work well in a closely bundled configuration when packaged appropriately within a soft, pliable, interstitial constraint to produce a high cumulative force output. In this fascicular arrangement, SPA packs also show improved robustness to failures induced by material rupture, blockage, or fatigue effects, as permitted by the advantage of multiplicity. With multiple independent active units comprising a single grouped SPA pack, the module as a whole is able to continue in operation with the consequence of unit failure limited to an inherent decrease in performance overall, and not a total loss of control. These features are critical for realizing safe and reliable high-performance soft robotic systems.
